# Subjective Well-Being and Schools in South Africa: A Post-COVID-19 Analysis

**DOI:** 10.3389/fpsyg.2022.891590

**Published:** 2022-06-22

**Authors:** Rommy Morales-Olivares, Carlos Aguirre-Nuñez, Lorena Nuñez-Carrasco, Felipe Ulloa-León

**Affiliations:** ^1^Department of Sociology, University of the Witwatersrand, Johannesburg, South Africa; ^2^Escuela de Construcción, Facultad de Arquitectura, Diseño y Construcción, Universidad de Las Américas, Santiago, Chile

**Keywords:** South Africa, COVID-19, school, material conditions, subjective well-being

## Abstract

From the analysis of the Wave 5 National Income Dynamics Study – Coronavirus Rapid Mobile Survey 2021 dataset, the study conducted in South Africa, we developed a model of analysis based on three dimensions, namely, subjective well-being, material living conditions, and importance attributed to education during the COVID-19 pandemic. A cross-sectional analysis of the data for Gauteng area indicates that the dimension of subjective well-being of families in South Africa—even in relation to the factors such as conditions of deprivation (e.g., hunger)—does not necessarily influence the importance the respondents attach to their children’s education, this as reflected in whether or not they send them to school when COVID-19 restrictions allowed for schools to come back to face-to-face teaching. Subjective well-being of parents and guardians is, however, a predictor of concern about their children’s education and future. Our working hypothesis is that, although there is little evidence that subjective well-being has a significant association with the respondents’ willingness for their children to continue their schooling, there is a significant indirect effect of subjective well-being—which is especially determined by the gender as well as of the living material conditions—and the greater or lesser importance that the respondents attribute to their children’s education. Likewise, and in more general terms, subjective well-being is clearly related to gender, with women having the lowest levels of subjective well-being.

## Introduction

The issue of education has been at the center of the national building project in South Africa in the past, during the apartheid, and continues to be the case till now in the democracy. During the apartheid, the Black population was forced into a purposely designed, bad-quality educational system. Known under the disparaging label of Bantu Education, this educational system aimed at reproducing the limited horizons available to Black people who were expected to perform as secondary class citizens in their segregated country (Christie and Collins, 1982). Furthermore, the languages spoken by most of the African population were subsidiary in the teaching and learning environments. Afrikaans was made the official medium of instruction which further disadvantaged and alienated African schoolchildren. It was during the Soweto youth uprising in 1976, when school children rebelled against the obligatory use of the Afrikaans language in the school, a poignant slogan “‘go to hell Afrikaans” read in one of the now famous placards held by these school children (Ndlovu, 1998; Bonner, 2004). Images of the brutal repression infringed on these school children caused widespread concern and helped mobilizing the international opinion against the oppressive system of the apartheid (Bonner, 2004). This was, as some analysts affirm, the beginning of the demise of the apartheid. The Soweto uprising also put at the center the role of education as a mechanism through which the segregationist nature of the apartheid system was reproduced (Ndlovu, 1998). In a democracy, the country’s educational system needed to be radically transformed not only to provide not only the formative support and abilities the new generation of “born free” South Africans required but also to provide the intellectual and critical skills the new nation needed.

Nelson Mandela’s newly inaugurated democratic government undertook the re-engineering of the educational system to overcome the multiplicity of disparities inherited from the apartheid system. While the transformations introduced by the democratic governments over almost three decades are significant, and their achievements are numerous, there are also many challenges that exist. The gap in educational outcomes and opportunities persist, and the access to resources and infrastructure is deeply unequal. This is still the case with rural and township schools which are schools in large Black population’s urban areas that were “grossly underserved and segregated by apartheid” (Ndimande, 2016). Educational inequalities have been reproduced along races, classes, and the urban–rural divide (Ndimande, 2016). With COVID-19, the goals and progress in education have been deeply affected. The measures implemented to respond to the COVID-19 pandemic, such as total and partial lockdowns, and the expected transition into online schooling has impacted on the most vulnerable households and on their children.

Based on the data set of the Wave 5 National Income Dynamics Study – Coronavirus Rapid Mobile Survey (NIDS-CRAM, 2021), this study takes a close look at households in the Gauteng province, the most populated in the country and home to the capital cities of Johannesburg and Pretoria, to explore how has COVID-19 impacted on school children. We focused on this region because it is part of the authors’ longer term research area. Gauteng has the highest number of ordinary independent schools (365) in the country and is the most diverse province in the country, in terms of racial groups and income levels. Of the approximately 14.6 million of learners at school in 2019 [Stats SA, 2020—South Africa’s General Household Survey (GHS)], the larger percentage attended schools in KwaZulu-Natal (21.8%) and in second place in Gauteng (19.7%). However, the percentages of learners attending the private schools is higher in Gauteng than in any other province with 13.6% of learner attending private schools and only 3.5% of learners in KwaZulu-Natal attending such institutions (Stats SA, 2020—South Africa’s GHS). Considering the diversity of Gauteng, we posed questions to the data set relative to the subjective well-being of parents and guardians in homes with school children in this province. While the questions we posed to the survey were not the ones that guided its design, it has allowed for an exploration of hypotheses and potential relations between the variables that we present in this study.

### South African Backgrounds

The new democratic government of Nelson Mandela promised socioeconomic justice and integration of the previously segregated and excluded Black population. The new constitution (1996) recognized equality, freedom, and human dignity for all people of South Africa. Affirmative action and the Black Economic Empowerment programs were put in place to revert the socioeconomic and political injustices of the past and undo the historical disadvantages faced by previously excluded groups. A central concern of the democratic governments has been the ending of the legacy of poverty and under-development, intended through the Reconstruction and Development Plan (RDP) of 1994 and in the National Development Plan (NDP) of 2011. However, the country has not succeeded in providing a better life for all, with an economic system that is still largely governed by neoliberal principles. With a Gini coefficient of 63 for 2014/2015 South Africa is one of the most unequal countries in the world (NIDS-CRAM, 2021). A considerable percentage of the population continues to live in poverty. Estimates for 2014/2015 show that 55.5% of the population live in poverty and 18.9% live below the poverty line (World Bank, 2020). Unemployment is persistently high and there is still a lack of access to housing, electricity, and piped water for the poor. Women are the most affected by the country’s history of exclusion and discrimination. Women are more impoverished than men; 58.6% are poor as compared to 54.9% for males (Stats SA). Women and girls continue to be victims of gender-based violence, mostly at home. According to the UN Women, in 2018, “13.1% of women aged 15–49 years reported that they had been subject to physical and/or sexual violence by a current or former intimate partner in the previous 12 months” (UN women). The patriarchal order of the society is reproduced through a rigid sexual division of labor in the households. The UN women reported for 2018 that “women and girls aged 10+ spend 15.6% of their time on unpaid care and domestic work, compared to 6.5% spent by men” (UN women). The raising of children is mostly women’s responsibility. The recent research has established that 60% of children in South Africa have absent fathers, and more than 40% of South African mothers are a single parent [Human Sciences Research Council (HSRC) and South African Race Relations Institute (SARRI), March 2020].

Despite these failures, many continue to expect the state to deliver on the promise of granting socioeconomic rights to all who remain excluded. South Africa, the rainbow nation, is a diverse society where the inequities of class, race, and gender have deepened with COVID-19.

### COVID-19 Situation in South Africa

Faced with the first cases of COVID-19 in South Africa, the government of President Cyril Ramaphosa declared a state of catastrophe and began the total confinement on March 27, 2020. A strict lockdown was implemented to buy time and prepare the health system as well as the general response to what seemed to be an imminent disaster. The closure of all ports of entry put an immediate end to international movements and inter-provincial transport. The prolonged lockdown has had devastating results for the poorest, for the women, and for the girls. The government’s response has been aimed at providing basic subsidies to the poorest and assisting them through an emergency COVID-19 subsidy to face the economic crisis resulting from the loss of jobs. The statistics of the third quarter of 2021 regarding the unemployment revealed the negative effects of the COVID-19 crises, as it reached 34.9%, the highest percentage ever (Stats SA, 2020). If a broad definition of unemployment is considered, one that includes those able to work but not looking for work, the percentage of unemployment then reaches 44%, affecting more than 11 million people. A figure that in the previous decades had reached 20%. The deepening of unemployment has impacted food insecurity. In 2017, 6.8 million South Africans experienced hunger (Stats SA, 2020). COVID-19 related measures such as lockdowns imposed additional burdens on low-income and food-insecure households.

The high level of for domestic, and public, violence. This was demonstrated by the events of July 2021, the eruption of unrest in the provinces of Gauteng and KwaZulu-Natal, two key economic centers of the country in which 354 lives were lost and thousands of businesses were looted and had to close. The unrest is estimated to have cost the country around 50 billion rand ($3.3 billion) in lost production, and put at risk at least 150,000 jobs, according to the South African Homeowners Association. Investigations have revealed that the violence was instigated by dissident groups within the African National Congress, the ruling party, to create fractures in the government.

It has been estimated that the deaths from COVID-19 have been underreported, being 3 times higher than the official numbers, as in March 2022, official figures reported a total of 302,960 deaths (SARMS, 2022) in a country with a population of around 55 million. Just as the likelihood that the excess death due to COVID-19 is underrepresented in the official figures, it can be said there is an invisible effect of the epidemic that will become evident in the post-pandemic period. South Africa has the highest number of people living with AIDS in the world, also the most massive anti-retroviral program, tuberculosis rates are also very high, largely a historical legacy of the precarious working conditions in the gold and diamond mines that enriched the country and made the nation the most industrialized in Africa. Several other chronic diseases have been neglected to respond to COVID-19. Thus, not only the chronic diseases but also the deterioration in mental health, an aspect that will become more visible in the future, and we explored it in our study.

Regarding vaccines, these began to be distributed in March 2021. To date, 18 million people have been fully vaccinated and 21 million have received at least one dose (Sacoronavirus, 2021). The vaccination process, although vaccination has been efficient, has met with significant resistance among people, including through anti-vaccine campaigns on social media. The possibility of establishing the obligatory nature of the vaccine is discussed, something that will have to be pronounced by the constitutional court, putting individual freedom and the protection of health of people as a public good in tension. This is how this COVID-19 crisis in South Africa has further opened the wounds of this country and it is not yet clear which process can strengthen the changes that are necessary.

In a country as unequal as South Africa, education, specifically, has a strategic value in overcoming the structural inequality determined by class, race, and gender (Ndimande, 2016). For the poor, accessing schools means also attaining food security. Through schools, families ensure that the basic needs, such as access to food, are met for their children. The challenge to achieve a more equal society includes the creation of a more inclusive educational system. A system that would embrace the diversity of languages, races, and cultures and simultaneously, is able to redress the gaps between rural–urban divide and create opportunities for the new generations to level the class abyss; hence is the importance of attending to the impact of the pandemic of COVID-19 on education.

In this study, we focus on the subjective well-being of parents and guardians in relation to the return of children to schools amid the COVID-19 pandemic. During the adjusted alert level in place from 1 March 2021 to 30 May 2021, most normal activities resumed including returning to schools. Subjective well-being, we consider, is closely related to the material conditions of households. In our analysis, the explanatory power of gender dimension emerges in relation to both subjective well-being and material conditions of the households. We consider subjective well-being as an intervening dimension in ensuring continuity in the education of children during pandemic and post-pandemic. School continuity, especially, has become complex in South Africa and percentages of out-of-school children have increased with the pandemic. While remote learning programs were designed, only the minority of schools had the necessary infrastructure that would secure access. According to a new report released by Statistics South Africa, “only 11.7% of schools offered remote learning options nationally. Most schools offered rotational options instead of remote learning and the urban–rural divide was prominent, as twice as many individuals were given the option of remote learning in urban areas compared to rural areas” (Stats SA, 2020). The disruptions that resulted from the closures of schools and rotational attendance had as an effect of reduced levels of attendance. As reported by Stats SA (2020), close to 1 million children were out of school; those aged 5–13 years were the largest number of out-of-school children. Country-wise, this age group was the highest in the Western Cape (13.0%), followed by KwaZulu-Natal (9.1%) (Stats SA, 2020). The cause of large percentages of out-of-school children was attributed to the COVID-19 pandemic because “parents/caregivers did not want to expose their children to the virus and subsequently kept them at home” and because most educational facilities closed due to COVID-19 (Stats SA, 2020). The impact of the pandemic might well mean a regression in the progress made over years in equalizing the quality of education and life opportunities for most of the Black population.

## Theoretical Framework: The Idea of Subjective Well-Being

The task of discussing and understanding the role of subjective well-being within the framework of the analysis of the development of countries, households, and individuals is relevant because according to the United Nations and its Human Development Programme (2012), it opens the opportunity to rethink progress by placing people at the center of the discussion on the objectives that a society should follow to be considered developed. Thus, addressing the issue of subjective well-being is a way to think about the development beyond mere economic growth, giving public and political relevance to other factors, such as mental and subjective health, security, gender, and trust in institutions, and also recognizing the fact that well-being cannot be separated from the social conditions that affect it.

Subjective well-being has traditionally been understood, from the field of psychology, as one of the relevant dimensions for determining quality of life. Another fundamental dimension is material or objective well-being (Moyano and Ramos, 2007) which, as an indicator, reveals an important information regarding the degree of life satisfaction with both material conditions of existence and influence that the future expectations have on personal happiness. This suggests that the social and relational conditions of existence and their influence on happiness are fundamental to understanding the degree of satisfaction of the population with their living conditions and through this comes the chances of having a physically and mentally healthy life that allows them to carry out life projects.

However, with regard to the discussion on the heuristic and political usefulness of the concept of Subjective Well-being (UNDP, 2012), there is a need to understand the idea of happiness by emphasizing not only its individual aspects but also its social determinants. This is tantamount to admitting that if subjectivity and its well-being are to be installed as an objective of development, it is necessary to give relevance to both, the degree of satisfaction and the expectations that people place on society and its institutions. This highlights the importance of issues that are traditionally outside the discussion on development, such as the concepts of social justice or the subjective feeling regarding the probabilities of success in personal projects *“(…) all subjectivity matters; not only that which refers to people’s view of their individual lives, but also to the image they have of society*” (UNDP, 2012, p. 30).

Such has been the problem of positive psychology, a current claim of psychology dedicated to the study of quality of life based on subjective well-being that emphasizes individual psychological characteristics. This is how Diener et al. (1999), relevant authors for the positive psychology, define it: “(…) *subjective well-being is a broad category of phenomena that includes people’s emotional responses, domains of satisfaction and global judgments of satisfaction with life*” (p. 277). This does not assign sufficient relevance to social factors and instead emphasizes the influence of individual determinants of well-being such as personality. This is problematic since individual satisfaction and subjective well-being do not only depend on the characteristics of individuals but also on the structural conditions of each society. This implies that through a proper understanding of how subjective well-being affects people’s lives, it is possible to develop better policies and to influence the solution of social problems.

More recently, Anand (2016) highlights two good reasons for countries to be interested in measures of well-being constructed from subjective appraisals, which is relevant for the construction of their public policies and the definition of their development goals. Anand (2016) points out that, first, the subjective measure of well-being is important because although the measure of income is an indicator of consumption, it does not fully serve to directly measure the quality of life. Second, he indicates that the values such as the quality of health, education, or gender equality are also an important part of well-being whose value, however, is not reflected in the indicators that have traditionally been used in economics to measure the development of countries. All of which suggests that well-being should be understood as a multi-dimensional phenomenon that is constructed from both subjective and objective factors, and its proper understanding has the potential to significantly affect the trajectory of individuals.

Now, following in this order of ideas, Anand (2016) highlights that, when addressing the subjectivity underlying the idea of subjective well-being, it is important to distinguish between satisfaction or pleasure, and fulfillment. He points out that an important part of subjective well-being can come from activities or responsibilities that are not pleasurable to exercise but are important in that they are directly linked to the possibilities of personal growth, self-acceptance, and the determination of life goals, giving as an example parenthood and the possibility of raising children according to self-determined standards. In this regard, Anand (2016) stresses that a decisive issue is the possibilities open to people to develop their potential, and as an example, it has been observed that gender equality is an important part of women’s autonomy and this directly affects social development through children’s education.

In the same vein, Anand (2016) indicates that there is a growing body of evidence showing that subjective well-being is connected to and directly affects personal health, people’s life expectancy, as well as the likelihood of success in education and in an education system. In support of this claim, Anand (2016) cites a study conducted in Bangladesh, Ecuador, India, Indonesia, Peru, and Uganda in 2006, entitled “Missing in Action: Teacher and Health Worker Absence in Developing Countries,” which concludes that the traditional objective indicators such as salary appear to have little impact on professionals’ attendance at educational and medical facilities, while the quality of infrastructure and its valuation did directly affect this.

### Education and Subjective Well-Being

With regard to the problem of education and its relationship with subjective well-being, UNICEF in collaboration with UNDP published a report in 2014 entitled “The Role of Education in the Formation of Subjective Well-being for Human Development,” reviewing the Chilean case, it concludes that the great challenge for education has to do with the possibility of acquiring the necessary tools to develop an autonomous life project. Education is a fundamental way of promoting the subjective well-being of the population from an early stage, but for this to be done properly, schools must not prioritize cognitive training over comprehensive training. In other words, it must be an instance where decision making and the acquisition of competencies for the development of life projects have a relevant place.

In the same vein, the UNICEF and UNDP (2014) highlights that, in the Chilean context, there is a strong criticism of the education system among students due to the gap between the expectations placed on the system and the assessment of how the school is meeting them. According to the study, students claim that, in addition to the skills that school should provide, which are in crises, and to those competences related to academic training, they demand tools for the development of their own life projects as well as the provision of training that makes students capable of participating and influencing society. This seems to indicate that a school is seen as an instance that should promote subjective well-being not only toward adulthood but also during school education itself. Although the study does not indicate how the school’s capacity to promote subjective well-being affects school attendance, it does provide relevant information in this regard, indirectly, by indicating that socio–cultural level is one of the determinants for the assessment of school from a subjective point of view. It indicates that students who are in a position of greater social marginalization tend to appreciate more what happens in the school space in terms of personal development. At the same time, however, students from higher socioeconomic strata, belonging to public schools, recognize that they have learned more in terms of these skills. This suggests that the expectations placed on the school system are also different between social classes and could eventually affect, if not the valuation of the school in particular, the importance or the degree of priority given to education in general. This places additional responsibility on the way in which the school as a social institution constructs the conditions for development.

Society and its institutions do matter, if they are decisive when weighing the degree of satisfaction of individuals with their lives. This means that subjective well-being is an area of work for development that should be at the center of the concerns of any country and its policies. As indicated by the UNDP: “[it is] possible and necessary to address the construction of the social conditions that allow all people to orient themselves toward their personal satisfaction; in other words, that society should use its resources to support people in achieving this objective” (2012, p. 50). It is only in this way that the life in the society enables citizens to recognize themselves and their everyday life in common experience and in public life. This is essential to understand the relationship between personal happiness and social well-being.

### Subjective Well-Being in South Africa

The question of subjective well-being and what it means in the context of each society varies according to the social conditions present and cultural particularities. This was indicated by Neff (2007), commenting that the high level of socioeconomic inequality in South Africa, part of the legacy of apartheid, is combined with a particularly relevant factor in the country, which is the origin and ethnicity of the different groups in society, which directly affects the possibility of fostering subjective well-being. As Neff (2007) points out, different ethnic groups in South Africa have different conceptions of what well-being means, and this affects how they assess their situation, even for members of the same racial (but not ethnic) groups, more significantly than the distribution of wealth.

Neff (2007) shows that, contrary to what might be believed, the historically privileged group of English-speaking White population, despite having the highest income concentration in South African society, is not the group with the highest level of subjective well-being. In the face of this fact, Neff suggests two possible hypotheses, not necessarily mutually exclusive. He points out that White people are a group that after the end of apartheid lost some of their legal and juridical protection, which could have affected their perception and optimism regarding the democratic transition, and led to a perception of a lack of security in all areas. Added to this is the possibility, suggested by the evidence, that high income, while helping to achieve high subjective well-being, after a certain point, ceases to have a significant influence on people’s perceptions of their situation. This is important because it shows that the relative position of each group directly affects their subjective well-being and the factors that are relevant for the promotion of a feeling of satisfaction and happiness. This is so even beyond socioeconomic indicators that reveal the material situation of each group. All of which puts the subjective development of the population; their psychosocial situation, and the role that institutions can play in helping to improve the level of satisfaction of the population, at the centre of the question.

Another good example of how institutions can directly influence the level of subjective well-being can be observed when analyzing how gender differences can influence the level of subjective well-being of the population. Fisher (2019) recently identified for South Africa that, until 2008, the level of happiness of men and women differed significantly, with men being much happier than women. He was also able to determine that one of the most important variables in determining this, in the case of women at that time, was being in a relationship and feeling satisfied with it. In the case of men, this had a very low influence. However, the relevant issue is that the same study was able to determine that by 2017, these differences between men and women had been significantly reduced, and the fact of being in a relationship had lost a significant part of its importance. Thus, the level of subjective well-being of men had increased moderately compared to 2007, but in the case of women, it had increased considerably.

The case of gender in South Africa, as Fisher (2019) explains, is relevant because it contradicts the global trend, especially in the so-called developed countries, where the level of subjective well-being of women is seen to be declining. It also proves that the policies promoting gender equality help to increase women’s level of happiness, while positively, or at least not negatively, influencing men’s level of subjective well-being. Likewise, the important differences between one period and another, observable not only at the level of the indicators but also with respect to the variables that are relevant when determining the level of subjective well-being, suggest that the determination of the level of subjective well-being of a population not only varies according to the values through which each group understands their happiness but also the importance of each factor varies over time, according to the position occupied within society. This makes it necessary to observe the particularity of each case, emphasizing the differences between the developed and the underdeveloped countries as well as the influence that the policies oriented toward the recognition of disadvantaged groups in society have had throughout in the recent history.

In a similar vein to that described above, Blaauw and Pretorius (2012) have identified that in South Africa, and possibly in other countries in the global south with similar problems, there is a great difference from what happens in first world countries, at least when it comes to assessing the subjective well-being of the population and, through that, people’s relative happiness and satisfaction. In conditions of material precariousness, insecurity, hunger, and deterioration of life in general, questions such as an individual’s high, state of health, and housing, although important, do not explain subjective well-being, as they do, at least in the case of South Africa, first, the cultural and religious influence, and then the territorial location of each group, with all these entail in terms of access to goods. This confirms the need to give different emphasis to the assessment of subjective well-being in different countries, depending on their level of development. Where the basic needs are relatively assured, Blaauw and Pretorius (2012) point out that subjective well-being is more related to individual issues related to appearance, health or access to goods, while in developing countries with significant material deprivation, low levels of security and high levels of inequality, factors such as race, ethnicity, marital status, gender, race, cultural identity, and access to basic services are determinants.

The difference in the assessment of subjective well-being between so-called developed and underdeveloped countries, and between the countries of the global south themselves and their regions, is most clearly evident when material deprivation reaches the extreme of food deprivation, hunger, as shown in our research. Food insecurity is an ongoing problem today and offers much important information about our understanding of subjective well-being. This is shown by Sulemana and James (2019), who study the relationship between food insecurity and indicators of subjective well-being in five sub-Saharan African countries. They conclude, as expected, that subjective well-being declines in countries where lack of access to food remains a problem. However, the same authors also conclude that the influence of food insecurity on subjective well-being is greater where access to food is occasional than when famine is so extreme that there is no assurance that it will be accessible in the near future. Likewise, Sulemana and James (2019) seem to observe that, when the situation reaches the extreme of lack of certainty regarding the possibility of being able to feed oneself the next day, the indicators that were shown to be relevant for the determination of the level of subjective well-being in other circumstances, such as gender, age, or employment status, lose importance. This makes it necessary, as Blaauw and Pretorius (2012) suggested, to consider the issue from a regional and territorial perspective.

However, a particularly relevant indicator, whose importance does not seem to disappear or diminish in the context of famine, at least according to Sulemana and James (2019), is education. Although in the context of food insecurity, unlike in other circumstances, higher levels of education are associated with lower levels of subjective well-being. According to the authors, this is explained by the high expectations placed on access to education, which, however, especially in this extreme situation of famine, is not enough to correct the difficulties and ensure access to food. This situation, however, although contradicting, as the same authors point out, the existing evidence on the relationship between education and subjective well-being, seems to be entirely consistent with the importance of education for people in underdeveloped countries.

This is observable if one considers the case of South Africa, where, as noted above, inequality is extremely deep. According to Neff (2007), in South Africa, the value associated with education is enormous because after the dismantling of apartheid and the installation of a democratic regime, the level of education became an important tool to establish the degree of success and the level of satisfaction of the population. Neff explains that part of the White population that saw its level of subjective well-being decline after the end of apartheid is a population that lost part of its racial privileges and was unable to regain them because its educational level, one of the new mechanisms for determining access to services and goods, was not high enough, and different policies were generated to horizontalize society.

Neff’s analysis is consistent with the observations of Blaauw and Pretorius (2012), who indicate that the one variable determining the level of subjective well-being whose importance does not vary significantly across South Africa, is education. According to the authors, the most educated individuals have the highest levels of subjective well-being, and each year of education completed contributes to and impacts the level of satisfaction of individuals. This, once again, highlights the decisive character of education in the question of human development. The importance that its promotion should have in the design of policies that seek to address the lack of satisfaction expressed by disadvantaged groups in society, whether for reasons of ethnicity, gender, race or territory. When the most basic needs are met, education seems to be a consistent way, at least as the evidence indicates, to redress social problems and their impact on people’s well-being. Even in an imperfect democracy with multiple problems such as South Africa, the importance of education, at least in terms of how people feel about their situation, is critical.

## Materials and Methods

The following section will explain the theoretical criteria on which the quantitative methodological work of the research was based. The analysis is composed of two fundamental methodologies, first, descriptive characterization of variables, which is the first approach to the sociodemographic, educational, and subjective well-being context of those living in the territory of Gauteng during the year 2021. Second, we developed two set analyses elaborated after a series of relevance tests, which is defined as a method of interdependence where we seek to group the sample in relation to their behavior in relation to educational variables and perception of subjective well-being. The relationship was then established between these subgroups and material factors grouped from a battery of survey questions. With this, all cases were grouped in the following two categories: Those who had sent their children to school and those who had not sent their children to school. Then, through a case selection, cases were grouped according to the gender of the respondent and whether they received benefits from institutions during the pandemic. The formation of these four groups is determined by the characterization of each one of them in relation to the factors associated with their perception and behavior regarding subjective well-being and elements associated with children’s education.

### Sample and Procedure

The questionnaire and material used for our analysis is based on the CRAM mobile survey, which is concerned with the social impact of the coronavirus pandemic in South Africa. This was conducted in May 2021 by telephone and participation was voluntary and consented by the respondents. The main characteristic of this type of statistical study is that it is of the panel type, which focuses on a problem and focuses on the evolution of this phenomenon over a period of time. Coronavirus Rapid Mobile Survey describes information on the same subjects from the beginning of the research until the end of its duration. The sample for this research consists of 473 people selected from households with school-aged children in the Gauteng province. This made up 53% of the total number of respondents in the province. Fifty-seven percent were female, while 43% were male. Gender, together with social and subjective well-being, is included as significant and representative variable in our analysis, and subsequent results that address the research objectives.

### Instruments

In the CRAM material used, the variables whose characteristics and nature corresponded to the qualitative order (ordinal and nominal) were generally selected, except for variables such as age, income in the month of March 2021, subsidies and the number of children attending schools before and after March 2021. A bivariate analyses of significance between variables and multivariate statistical analyses were carried out to synthesize the interrelationships observed between the set of variables selected for this study.

### Descriptive Analysis

A series of descriptive data emerged from the question related to the state or perception of the mental health of the residents of Gauteng after the appearance of the COVID-19 pandemic that allows us, in the first instance, to describe the subjective state of the respondents, and to understand this in relation to their sociodemographic, educational, and economic factors. It should be noted that only 53.5% of those surveyed in Gauteng have completed their primary school studies. In March 2021, 87.3% of the survey respondents in Gauteng had children attending school. From the beginning of the pandemic, from March 2020 to March 2021, the average number of children attending school only decreased from 1.69 to 1.59 children per household in this province. This percentage is smaller than the above-mentioned dropout rate.

Almost all children have returned to school, and a number of variables are related to this. A total percentage of 42.7% of people consider themselves to be very concerned about the return of children to their academic activities, and 43.5% of people whose children receive school meals are similarly concerned. By performing Pearson’s Chi-squared tests, it was possible to establish that there is a significant incidence in the degree of concern for the return to school closely related to the variable of homes where minors have suffered from hunger in the last 2 weeks. This is due to the fact that the *sig.* 0.005 (i.e., *p* < 0.05) rejects H0 and assumes affirmatively the veracity of H1. Another factor considered is mental health. In relation to the variables associated with the return to school of school students. Among those who have felt depressed almost all the time in the last 2 weeks of March 2021, 58.5% are worried about the children’s return to school. The analysis shows that the more depressed they have felt, the higher the percentage of concern about children returning to school. From a gender perspective, those who have worse mental health are in most cases women, since 68.3% of the people who declare to have poor mental health are women, who are presumably caring for minors. All these are composed as elements extracted from the variables of significant relevance in the return to school of most of the children in Gauteng.

Finally, the data and the variables from the survey are relevant to conduct a univariate analysis that allowed to describe the situation in Gauteng in May 2021 (Table 1).

**TABLE 1:** Descriptive approach to the situation in Gauteng in May 2021.

Gender (%)	
Men	45.4
Woman	54.6
**What type of area are you living in now? (%)**	
Traditional area/chiefdom	5.6
Urban area/town	84.8
Farm/rural area	7.8
**Number of people resident, including yourself (don’t forget babies)**	
Average	4.30
**In March, which of these was the main household income source? (%)**	
Income from employment	52.7
Income from a business	4.8
Government grants	28.2
Money from friends or family	8.1
Household had no income in March	0.6
Pension	5.3
**Own elaboration/CRAM survey**	
**Number of children attending school before school closures in March 2020**	
Average	1.69
**In the past 2 weeks, how many young people in your household attended school?**	
Average	1.58
**How worried are you about household learners returning to school? (%)**	
Not worried	19.9
A little worried	37.4
Very worried	42.7
**In the past 2 weeks, have any household learners received a meal from school? %**	
Yes	38.0
No	49.3
Not applicable “No young people at school”	12.7
**Own elaboration/CRAM survey**	

### First Set Export

Initially, the study aimed at conducting a factor analysis, which according to Cea (2002) consists of a work of reduction of variables to identify the formation of factors that explain most of the variance of a specific number of selected variables. Given the condition of the survey questions and the failed Kaiser, Meyer y Olkin (KMO) test (0.38), the methodological decision was to reverse the work and develop two groups of analysis, to strengthen the statistical analysis. The first step focused on developing niches which were divided between the households that did/did not send children to school at the end of the lockdown in March 2021 after the health crisis caused by COVID-19. This methodological decision strengthened the statistical analyses and provided us with a pattern of behavior for those households with children attending schools.

A decision was made to make a case selection regarding households where children had or had not returned to school by March 2021. For this purpose, statistical tests of the cross-table and ANOVA type were made. This was to see if there was a relevant significance in the variability of the variables of return to school with different variables. This would allow us to make a more complex analysis on the research problem which is linked to subjective well-being. One of the first subsequent steps was to the groups of the households where children had returned to school correspond to 90% of the respondents while only 10% of them had not returned to school.

In this situation, it is important to understand the behavior of both groups. Especially those in which the children did not return to school. In view of this, it can be said that there is a real significant coincidence in the return to school of children and the respondents’ concern about the return to school. Indeed, among the total number of respondents who felt very concerned about the return of children to school, 88% respondents had their children back in the educational system in March 2021. These figures are accompanied and complemented by subsidies that families received or requested during the pandemic, such as Covid’s Special Distress Relief Grant, from the government during the pandemic, food and shelter assistance from the government and/or non-governmental organizations. In all of these categories, those who sent children to school have the greatest involvement in applying for and receiving grants.

Then, another point of interest for the study is the perceptions of material precariousness and discomfort in their own perception of their health experienced by the respondents. In relation to material precariousness, we refer to the problems associated with food insecurity in households where children have or have not attended school during the month of March 2021. The data indicate that 82.9% of the people who had children suffering from hunger in their homes sent their children to school, with a justified significance in the results of the significance in the Chi-squared test. To this, we add the variable of income for the month of March 2021, since this as it was established, which is a variable on which the variability of other subjective variables on the perception of one’s own well-being depends, such as the following:

• g16_How would you describe your health at present?
• g1_Do you think you are likely to get the coronavirus?
• ga19_Over the last 2 weeks, have you had little interest in doing things?
• ga20_Over the last 2 weeks, have you been feeling down, depressed, or hopeless?

On the other hand, there are conditions and perceptions about one’s own health that we also take as relevant variables when talking about people’s subjective well-being. Regarding the perceptions of mental health and mood in the last 2 weeks, we can affirm that most of the people who feel unwell in relation to their mental health send their children to school. An example of this is that 93.3% of the people who feel depressed almost every day in the last 2 weeks send their children to school, or that 58.5% of the same total of people felt very worried about the return of school classes. These relationships between mental health and sending children to school continue to be repeated in health variables such as the perception of their current health, where the percentages of children returning to school continue to be higher the worse the perception of their own health.

### Second Exploration in the Analysis

Once the descriptive statistical analysis and the first exploration of sets have been carried out, it is convenient to establish the last dimension of analysis. Given that it is not useful to have one group composed of 90% of the sample and another with 10% (those who sent and those who did not send their children to school), we chose a new dimension of analysis corresponding to the variables nested in groups that are segmented according to the descriptive variables relevant for our study. A battery of variables was identified to establish basic elements of subjective well-being. We coded the variables into a single variable, which allowed us to group all people who had received subsidies from any institution as well as those who had not. The rest were kept as they were in the survey to work with them to establish basic descriptive statistics in each of the niches we have identified. The analysis of the selected dimensions allows to establish the main differences between the respondents, also allows to visualise the relation between material condition of the household and subjective well-being as well as the respondents perception of children‘s education. These dimensions are presented in Table 2 (Subjective well-being), Table 3 (Material condition of the household), and Table 4 (Education).

**TABLE 2:** Subjective well-being.

	Men	Women
With subsidies	(+; +) Only 35% of them have the feeling that they will catch COVID; Only 4.8% of them have a poor perception of their current health and 42.9% say they are stable; 66.7% of them say they have not felt a loss of interest in doing things; 71.4% of them say they have not felt depressed at any time in the last 2 weeks	(–; +) 89.1 % of them stated that they have not had underage children in hunger during the last 7 days; 51.1% of them believe they will have COVID; 27.5% of them say they are in reasonable health, while 13.7% of them say they are in poor health; 18% of them say they have not lost interest in doing things during the last 2 weeks; 13.7% of them say they felt depressed every day of the last 2 weeks, while 52.9% did not feel that way on any day; 13.7% of them say they felt depressed every day of the last 2 weeks, while 52.9% of them did not feel that way
Without subsidies	(+; –) 40.6% of them have children who have been hungry during the last 7 days; Only 34.8% of them believe that they will have COVID; 31.5% of them say they are in good health at present and 9.3% of them say they are in bad health; 25.9% of them say they have lost interest in doing things during the last 2 weeks; 59% of them say they have not felt depressed during the last 2 weeks, while 13% of them say they have felt depressed every day	(–; –) 23.3% of them have had hungry children in their homes during the last 7 days; Only 30.8% of them believe they will have COVID; 12.1% of them say they are in poor health at present; 24.1% of them say they have lost interest in doing things during the last two weeks; 8.8% of them say they have felt depressed during the last 2 weeks

**TABLE 3:** Material condition of the household.

	Men	Women
With subsidies	(+;+) 92.5% of them have access to drinking water in their homes; 85.7% have access to electricity in their homes; 42.9% of them reported having run out of money for food during the month of March	(–;+) 94.1% have access to drinking water in their homes; The same 94.1% have access to electricity in their homes; 49% of them stated that they ran out of money for food in the month of March 2021
Without subsidies	(+;–) 11.1% of them do not have access to drinking water in their homes; 20.4% of them do not have access to electricity in their homes; 46.3% of them ran out of money for food during the month of March	(–;–) 91.4% of them have access to drinking water in their homes; The same 91.4% have access to electricity in their homes; 56.9% of them stated that they ran out of money for food in the month of March

**TABLE 4:** Education.

	Men	Women
With subsidies	(+;+) Less than half of them completed their schooling (42.9%); 9.1% of the children in their homes did not return to school; High % of children feeding in schools (54.5%); 50% of them are really concerned about the return of children to schools	(–;+) 52.9% of these households completed their schooling, but do not have people who have had access to higher education; 10.9% of the households have children who have not returned to school; only 33.3% of the children in these households receive school meals; 65% of these households are very concerned about the return of their children to school
Without subsidies	(+;–) 38.9% of them completed their schooling; 93.8% of the group households have their children back in school; 51.7% of them have their children receiving school meals; Less than half of them were worried about going back to school (48.3%)	(–;–) 55.2% of them stated that they had completed their schooling; 93% of the households had their children back in school; 57.1% of them had their children being fed in school; 61.1% of them were very worried about going back to school

For the creation of the groups, a new variable was created from a battery of variables present in following questions:

– cg6 – Did you receive any Unemployment Insurance Fund (UIF) benefit in March?
– da6 – Did you apply for the special COVID-19 relief from distress grant?
– da8 – Did you personally reecive any kind of government grant in March?
– dc1 – During March, did you receive food or shelter from the government?
– dc2 – During March, did you receive food or shelter from any non-governmental organization (NGO)/church?
– dc3 – During March, did you receive food or shelter from the community?

**FIGURE 1:**
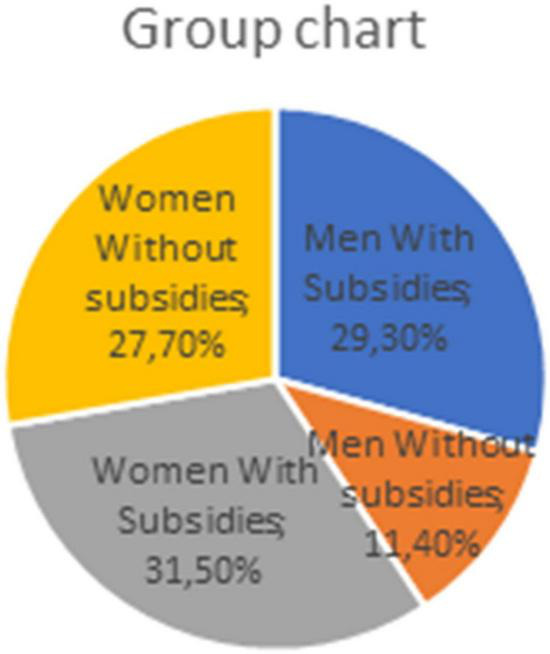
Own elaboration/Wave 5 CRAM survey.

Out this new variable “receiving help from institutions,” four groups were formed (Figure 1):

– Men with subsidies (+; +) = 29.3%.
– Men without subsidies (+; –) = 11.4%.
– Women with subsidies (–; +) = 31.5%.
– Women without subsidies (–;–) = 27.7%.

In Table 2, it can be seen how the group of men presents similar conditions regarding their perceptions of subjective well-being. It is the group “without subsidies” the one that presents a slight decrease in subjective well-being, so it can be inferred a negative impact of the lack of subsidies on wellbeing. In Table 3, in the case of the two groups of men shows that the housing conditions of those who do not receive subsidies are worse than those who do. Finally, Table 4 also shows the percentages of respondents that express concern for the children’s return to school are similar and low.

On the other hand, there are two groups of women that are differentiated in the same way as men, i.e. in terms of whether or not they received subsidies from private and public organisations during the pandemic. Table 2, shows that women have a higher percentage of concern about Covid infection, hence gender appears to be a key element in understanding the variation in subjective well-being. Women have worst subjective well-being as compared to me, and this percentage is even worst in the group that does not receive subsidies during the time of the pandemic. Table 3 shows that while female respondents’ households have better material conditions than men’s respondents, their households have the highest number of people without money for food during the month of March. Finally, Table 4 shows that women are the most concerned about the return of children to school after the lockdown in South Africa.

### Gender Perspective

After observing the behaviour of the gender variable in the analysis (Tables 2–4), it is clear that being a woman introduces a greater weight in the perception of subjective well-being. It is because of this, that a methodological decision was made to, a methodological decision was to take gender as an independent variable; its statistical significance is present in the variability of all the other variables used in the study. We conducted an analysis of crosses of variables dependent on gender. The results show that those respondents that have the worst perception of their mental health are in most cases women (68.3%). It was mostly women who were more worried about the children’s return to school; hence, there is a relationship where worse mental health is declared, the higher the percentages of concern about the children’s return to school.

In a context in which children were out of regular school attendance due to the various regulations associated to COVID-19, the inclusion of variables referring to child care was taken as a relevant factor. After crossing the variable of gender with the variable on who performs childcare tasks, we could see that it is women who perform the role of caregivers in households and it is men who hire the most services for childcare. Finally, the economic condition is also configured as a strong variable with respect to subjective well-being, captured as the income perceived in the month of March. While it is often understood that the more income people have, the better perception they will have with respect to their mental and general health. After analyzing the descriptive data on income by gender, we can also see that women have a much lower average income than men during the month of March.

## Conclusion

First, we should recall that analysis of the Wave 5 National Income Dynamics Study – Coronavirus Rapid Mobile Survey (NIDS-CRAM, 2021) dataset in South Africa has been conducted in a cross-sectional manner. The survey has been conducted for a different purpose and all our analysis has been conducted on the basis of a comprehensive systematization of secondary data, the NIDS-CRAM (2021) is not a subjective well-being survey. The exploration of the survey has been carried out with an initial hypothesis concerning the relationship between subjective well-being and concern about education, which has been partially corroborated for the case of people who feel concern about education. The lower the subjective well-being, the greater the concern about education. In summary, from a trial-and-error analysis in the construction of our model, we have developed a derivative for which the study was not necessarily designed, but which proves the relevance of the application of our hypotheses and theoretical framework as a prism of observation.

Second, it is important to mention that the vast majority of people in the sample send their children to school after the change in COVID-19 regulations, so it can be said that sending or not sending children to school is not an indicator of distinctions in relation to subjective well-being or material conditions, since 9 out of 10 people send their children to school regardless. However, the proportion of people who did not send their children to school tend to have better material living conditions one could hypothetically infer that they have better conditions to provide educational conditions at home and care, but this hypothesis cannot be corroborated since there is no data in the survey to do so.

Third, and in line with the above, the concern for education and sending children to school do not have a symmetrical relationship. In other words, even if there is not a high concern for education, families still send their children to school. In this sense, the school stands as a security device for families and an institution that provides other elements that do not necessarily have to do with educational aspects, but with subsistence aspects.

Finally, and in relation to subjective well-being, there is a clear line drawn between being a woman and having poorer subjective well-being. This reality is not specific to South Africa or to the data analyzed; there is already an important body of literature (some of which is shown in the theoretical section) which gives an account of this reality. However, it is particularly worrying for the case in question, considering that the variables included in our definition of subjective well-being are related to mental health. Similarly, the material conditions of existence are also worse for Gauteng women. Considering the two variables together reveals a persistent problem: the precariousness of women’s lives regardless of the context or situation of crisis, which worsens with the crisis. In addition, the mental burden of worrying about education falls more heavily on women, who devote more time to child care and are more fearful about the uncertain future in the context of the COVID-19 pandemic.

The limitations of this study is that the analysis only includes the data for Gauteng, while it would have been a more robust analysis had we included all information available in the survey, country wide. This decision was based on the interest of the research team to produce knowledge for Gauteng, an area where they have a specific research focus.

## Data Availability Statement

The original contributions presented in this study are included in the article/Supplementary Material, further inquiries can be directed to the corresponding author/s.

## Ethics Statement

Ethical review and approval were not required for the study on human participants in accordance with the local legislation and institutional requirements. Written informed consent from the patients/participants or patients/participants legal guardian/next of kin was not required to participate in this study in accordance with the national legislation and the institutional requirements.

## Author Contributions

All authors listed have made a substantial, direct, and intellectual contribution to the work, and approved it for publication.

## Conflict of Interest

The authors declare that the research was conducted in the absence of any commercial or financial relationships that could be construed as a potential conflict of interest.

## Publisher’s Note

All claims expressed in this article are solely those of the authors and do not necessarily represent those of their affiliated organizations, or those of the publisher, the editors and the reviewers. Any product that may be evaluated in this article, or claim that may be made by its manufacturer, is not guaranteed or endorsed by the publisher.
