# Supplementary figures and images for: Subjective Well-Being and Schools in South Africa: A Post-COVID-19 Analysis
Source: Front Psychol. 2022 Jun 22;13:891590. doi: 10.3389/fpsyg.2022.891590 (PMC9257172; doi:10.3389/fpsyg.2022.891590)

**Annexes**


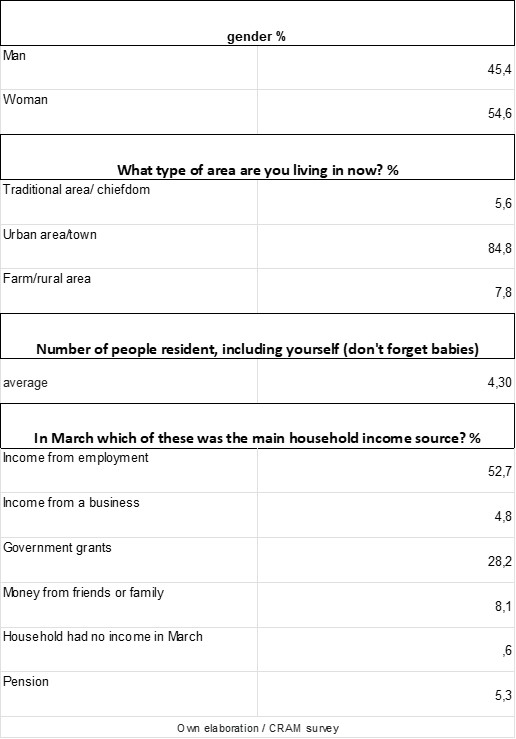


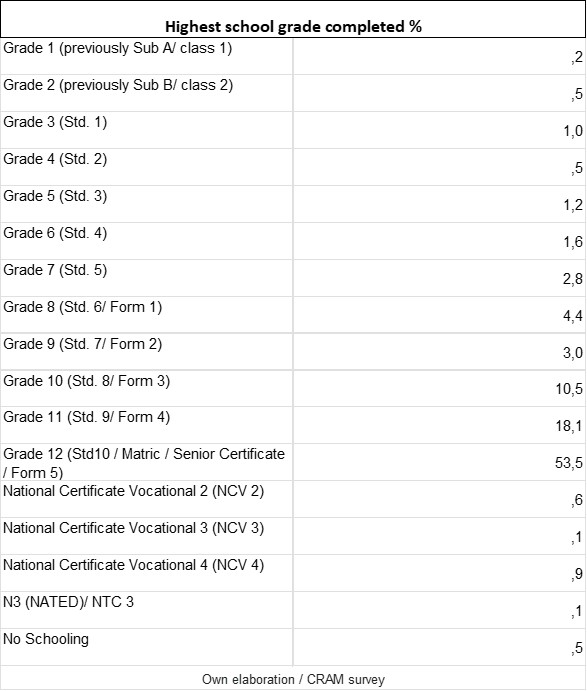


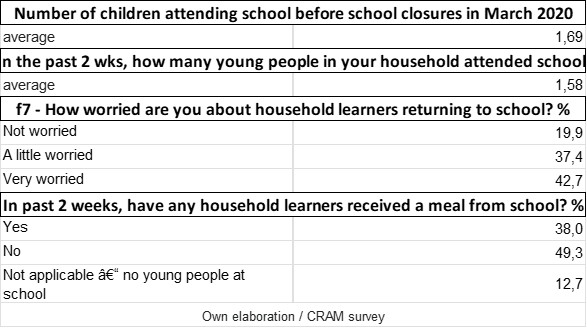


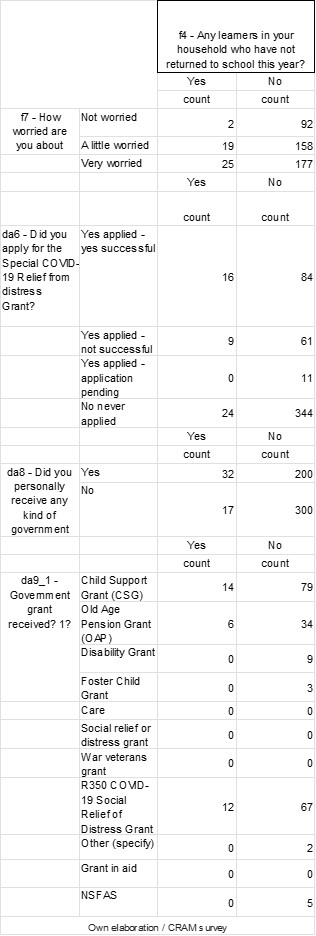


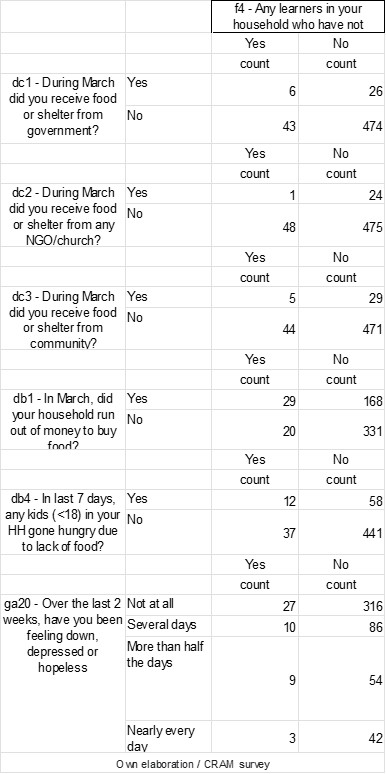


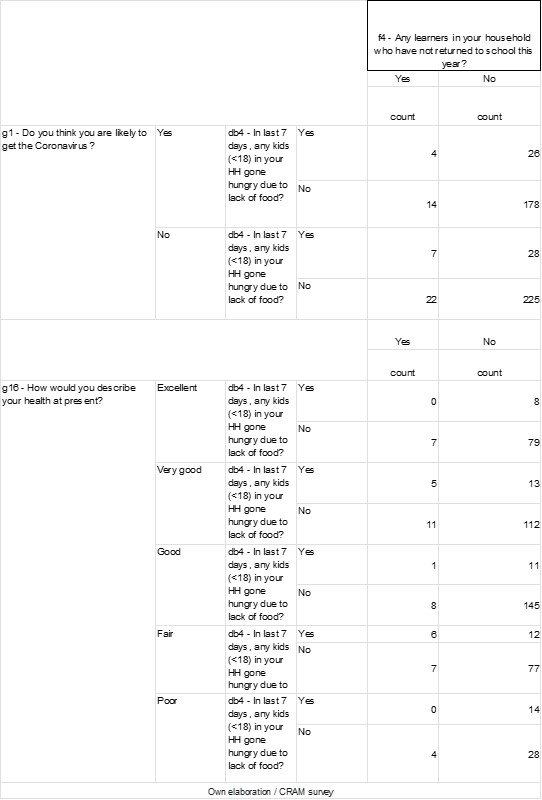


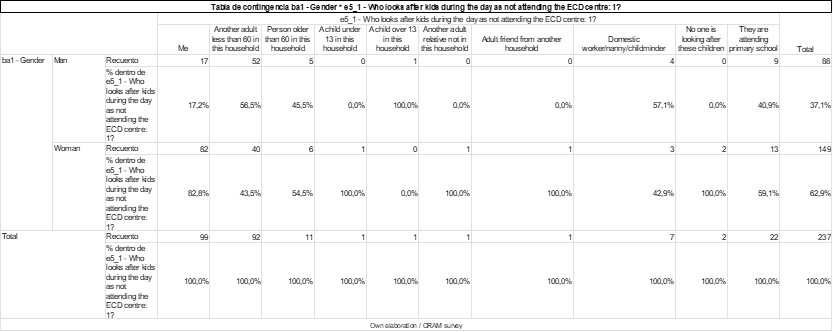

Supplement: Supplementary file 1 [file Table_1.DOCX]
